# Neural contributors to trauma resilience: a review of longitudinal neuroimaging studies

**DOI:** 10.1038/s41398-021-01633-y

**Published:** 2021-10-05

**Authors:** Alyssa R. Roeckner, Katelyn I. Oliver, Lauren A. M. Lebois, Sanne J. H. van Rooij, Jennifer S. Stevens

**Affiliations:** 1 Department of Psychiatry and Behavioral Sciences, Emory University, Atlanta, GA USA; 2 Division of Depression and Anxiety Disorders, McLean Hospital, Belmont, MA USA; 3 Department of Psychiatry, Harvard Medical School, Boston, MA USA

**Keywords:** Predictive markers, Psychiatric disorders, Neuroscience

## Abstract

Resilience in the face of major life stressors is changeable over time and with experience. Accordingly, differing sets of neurobiological factors may contribute to an adaptive stress response before, during, and after the stressor. Longitudinal studies are therefore particularly effective in answering questions about the determinants of resilience. Here we provide an overview of the rapidly-growing body of longitudinal neuroimaging research on stress resilience. Despite lingering gaps and limitations, these studies are beginning to reveal individual differences in neural circuit structure and function that appear protective against the emergence of future psychopathology following a major life stressor. Here we outline a neural circuit model of resilience to trauma. Specifically, pre-trauma biomarkers of resilience show that an ability to modulate activity within threat and salience networks predicts fewer stress-related symptoms. In contrast, early post-trauma biomarkers of subsequent resilience or recovery show a more complex pattern, spanning a number of major circuits including attention and cognitive control networks as well as primary sensory cortices. This novel synthesis suggests stress resilience may be scaffolded by stable individual differences in the processing of threat cues, and further buttressed by post-trauma adaptations to the stressor that encompass multiple mechanisms and circuits. More attention and resources supporting this work will inform the targets and timing of mechanistic resilience-boosting interventions.

## Introduction

Resilience has been defined as the set of complex and dynamic processes that allow individuals to maintain psychological well-being in the face of adversity [1, 2]. By this conception, resilience is not merely the absence of psychopathology, but involves a number of processes that can change over time. These dynamic processes are nowhere more apparent than in the psychological response to trauma exposure. Cohort studies tracing symptom levels from hours to years post-trauma show that many people initially experience high levels of depression, anxiety, and posttraumatic stress disorder (PTSD) symptoms in the first days after a traumatic event, but the majority recover naturally [3–5]. Therefore resilience does not provide a blanket protection against symptoms. Instead it is manifested in the capacity to recover to a state of well-being within a few weeks to months of a major stressful life event. Notably, resilience is the most common response to a traumatic event, as only a minority of individuals go on to maintain persistent impairing symptoms [4].

Neuroimaging data collected from before a traumatic event, during the peri-trauma, and during the post-trauma period can show the full sequence of neural features and adaptations that lead to recovery. Understanding neural circuit contributors to an adaptive response to trauma is critical to our ability to create new interventions that will improve and accelerate recovery. This is in line with current research priorities of the National Institute of Mental Health, which underscores the need for “*the development of translatable biomarkers [to] facilitate the study of stress responses, resilience, and vulnerability across both human and animal studies* [6]*”* *.* To facilitate the development of a longitudinal model of neural contributors to resilience, here we review the emerging body of literature employing neuroimaging in longitudinal cohort studies to predict psychological well-being following trauma exposure. For the purposes of the review, trauma is defined as an event meeting DSM-5 criterion A for trauma- and stressor-related disorders [7] such that participants experienced, witnessed, or were confronted with the threat of death or serious injury or threat to personal integrity. This definition was expanded to consider studies of other major life stressors, such as the death of a family member, job loss, or medical illness. Such stressful life events may elicit similar neurocognitive adaptations as “criterion A” traumas, a topic of active investigation. Finally, because resilience is not merely the absence of a psychiatric diagnosis, where possible, we highlight findings that show neural predictors of multidimensional aspects of well-being following trauma or other major stressor.

We conducted a literature review of neuroimaging studies from January 1995 to May 2021, using the following search terms: trauma OR PTSD OR “posttraumatic stress disorder” AND prospective OR longitudinal OR cohort OR predict AND MRI OR neuroimaging OR “magnetic resonance imaging” OR “neural correlates”. This search was conducted in Pubmed, Web of Science, and Scopus. We included all unique studies that had a longitudinal component such that neuroimaging was conducted either at one or multiple timepoints, with subsequent assessments of trauma-related symptoms including posttraumatic stress disorder (PTSD) or depression, and excluded findings from drug or intervention studies. Table 1 contains a full list of the longitudinal neuroimaging studies of trauma.

**Table 1:** Longitudinal neuroimaging studies of trauma exposure.

Paper	*N*	Breakdown TE = trauma exposure; TEC = trauma exposed control; HC = healthy control	% Female	Age (years) M ± SD (range)	Trauma type	Time since trauma + delay to follow-up	Structural differences	Functional differences	Connectivity differences
Bonne et al. [111]	37	All TE, 10 dev. PTSD	51	30.9 (20–65)	Mixed - ED	1 wk + 6 mo.	Hippocampal volumes (no difference)	x	x
De Bellis et al. [112]	18	9 maltreated, 9 HC	44	10.6 ± 13.1 (9–14)	Childhood maltreatment	1–5 yrs + 2 yr	Temporal lobe, amygdala and hippocampal volumes (no difference)	x	x
Hakamata et al. [66]	184	14 PTSD, 100 TEC, 70 HC	100	46.6 (18–55)	Cancer-related	varied + 2 yrs	Orbitofrontal cortex (larger in resilience)	x	x
Admon et al. [16]	62	50 TE soldiers, 12 HC civilians	50	18–19	Military deployment	Baseline (pre-deployment) + 18 mo (post)	x	Hippocampal (less change in resilience) and amygdala (decreased in resilience) reactivity	vmPFC-Hippocampus (greater in resilience)
Lanius et al. [62]	11	All TE	55	37	Automobile or Workplace accident	6 or 12 wks	x	x	PCC - pgACC, PCC - rAmygdala (both weaker in resilience)
Cardenas et al. [78]	47	25 PTSD, 22 HC	0	51.2 (33–60)	Military + non-military	~33 yrs + 2 yrs	Brainstem, frontal, and temporal lobe (less atrophy in resilience)	x	x
Daniels et al. [117]	20	All PTSD	85	36.4 ± 12.5	Mixed - ED	2–4 mo	x	(Right): cuneus, lingual gyrus, inferior and middle occipital gyrus, superior temporal gyrus; (Left): putamen, pre and post-central gyrus, transverse temporal gyrus, SN, mGP (less activation in all areas associated with less cog. distortion)	x
Dickie et al. [59]	18	All PTSD	72	36.8 (20–60)	Mixed	varied + 6–9 mo	x	Amygdala, vmPFC (decrease in resilience), hippocampus, sgACC (increase in recovery)	x
Lyoo et al. [89]	66	30 TE, 36 HC	62	26.7 (18–50)	Subway disaster	1.42 + 2.61 + 3.85 yrs	dlPFC (increased in resilience)	x	x
Papagni et al. [35]	26	All healthy	50	25.2	Stressful life events (SLEs)	Baseline + 3 mo	Left parahipocampal, right hippocampal, and bilateral ACC grey matter volumes (increased in resilience)	x	x
van Wingen et al. [22]	57	32 combat exposed, 25 HC	5	24.1	Military deployment	Baseline (pre-deployment) + 1.5 mo (post)	No significance	Amygdala & Insula (increased pre- to post-)	Amygdala - Insula, Amygdala - dACC (decreased in resilience)
Daniels et al. [95]	21	All PTSD	81	38.2 ± 12.1	Mixed - ED	2–4 mo	x	Ring lingual, fusiform, and parahippocampal gyri (negatively correlated with resilience)	x
Daniels et al. [91]	70	All PTSD (N = 12 for MRI)	59	36.2 ± 12.6	mixed - ED	2–4 mo	x	Right thalamus, inferior and middle frontal gyri (increased in resilience)	x
Van Wingen et al. [23]	39	23 deployed, 16 non-deployed soldiers	5	23.7	Military deployment	Baseline (pre-deployment) + ~1.6 mo + ~22.7 mo	x	Amygdala (returned to pre-deployment levels at long-term timepoint)	Amygdala - dACC (decreased in resilience)
Admon et al. [17]	24	All combat exposed	50	18 (18)	Military deployment	Baseline (pre-deployment) + 18 mo (post)	x	Amygdala (decreased in resilience) & NAcc (increased in resilience)	x
Dickie et al. [47]	30	All PTSD	67	36.4 (20–60)	Mixed	6–61 wks + 22–53 wks	sgACC (increased in resilience)	x	x
Sekiguchi et al. [27]	42	All TE	21	21.7	Natural disaster	~9.1 mo pre-trauma + 3–4 mo post	Right vACC (higher pre-trauma correlated with resilience) & left OFC (higher post-trauma correlated with resilience)	x	x
Sun et al. [49]	60	All TE, 21 dev. PTSD	53	~38 (18–60)	motor vehicle collision	2 days + 1 or 6 mo	ACC, vmPFC, temporal lobes, and midbrain (increased FA in resilience)	x	x
Weems et al. [118]	48	24 TE, 24 healthy	42	10.96 (7–14)	Mixed	Varied + 12–18 mo	Amygdala (smaller in resilence)	x	x
Gong et al. [75]	150	50 PTSD, 50 TECs, 50 Healthy	64	42.76 ± 10.6	Natural disaster	~1 year	Grey matter differences distinguish resilience	x	x
McLaughlin et al. [15]	15	All TE	66.7	16.5 (14.1–19.1)	Terrorist attack	~1 year pre-trauma (2–60 weeks before event)	x	Amygdala (decreased in resilience) & Hippocampus (increased in resilience)	x
Sekiguchi et al. [28]	30	All TE	6	21.0 ± 1.6	Natural disaster	~9.1 mo pre-trauma + 3–4 mo post	Right anterior Cg FA (greater in resilience pre-trauma). left anterior Cg and Uf FA (less change in resilience)	x	x
Sekiguchi et al. [37]	25	All TE	24	21.7 ± 1.4	Natural disaster	~9.1 mo pre-trauma + 3–4 mo post + ~1 yr	Right anterior Cg, bilateral Uf, left SLF, and left thalamus (less change in resilience)	x	x
Du et al. [119]	42	21 TE, 21 HC	38	39.1 ± 11.1	Natural disaster	3 wks + 2 yrs	Grey matter or white matter (no differences)	x	Frontal–limbic–striatal connectivity (recovery associated with return to baseline)
Reynaud et al. [34]	12	2 TE, 10 HC	0	21.4 ± 1.7	Not specified	Pre-exposure + 1 week	x	Right Amygdala, right OFC, right dlPFC, and BA9 (decreased in resilience)	x
Roy et al. [63]	81	All combat exposed	13.6	29.7 ± 7.9	Military deployment	2 mo post-deployment	Right SLF volume (greater in resilience)	x	Right amygdala - left superiortemporal gyrus rsFC (decreased in resilience)
Swartz et al. [19]	340	All healthy	57	20.8 ± 1.5 (18–26)	Mixed/stress	Baseline (anytime)	x	Bilateral amygdala reactivity (decreased in resilience)	x
Banks et al. [81]	24	13 mTBI, 11 HC	31, 36	39.3, 37.6	Mixed	6 weeks + 4 mo	x	x	Thalamus-dorsal attention network connectivity (increased in recovery)
Cwik et al. [79]	19	All TE (ASD)	74	33.5 ± 12.2	Mixed	<4 weeks	x	Right medial precuneus, lef RSC, precentral and right superior temporal gyrus reactivity (decrease in resilience); lateral, superior prefrontal, and left fusiform gyrus activation (increased in resilience)	x
Hu et al. [86]	34	PTSD, TEC	50	42.18 ± 12.07 (PTSD), 38.59 ± 13.2 (TEC)	Motor vehicle collision	2 days	Anterior thalamic radiation, cortico-spinal tract, forceps minor, uncinate, inferior fronto-occipital fasciculus, ILF, cingulum and SLC FA (greater in resilience)	x	x
Ke et al. [51]	28	19 acute PTSD, 9 TEC	0	34.5 ± 4.7 (PTSD), 39.2 ± 5.3 (TEC)	Mining accident	2 mo + 2 yrs	x	mPFC and inferior parietal lobules (greater in resilience pre-trauma); right middle frontal gyrus, PCC/precuneus, vermis and cerebellum activation (decreased in resilience)	x
Li et al. [120]	65	43 mTBI (22 successful recovery, 21 poor recovery), 22 HC	45	35.8 ± 7.58 (mTBI recovery), 36.7 ± 7.09 (mTBI poor recovery), 36.1 ± 7.11 (HC)	Traumatic brain injury	3 days + 10–20 days + 1–6 months	Greater FA and lower MD associated with recovery.	x	x
Nilsen et al. [64]	40	23 TE, 17 HC	25	40.2 ± 12.5 (TE), 37.1 ± 9.6 (HC)	Motor vehicle collision	3 wks		Occipital cortex, temporal cortex, thalamus, frontal and superior parietal area activity (greater in TE group)	Amygdala-somatosensory connectivity (increased in trauma-exposure)
Wang et al. [46]	44	21 mTBI, 23 TEC	59	34.3 ± 11.2 (mTBI), 33.8 ± 11 (TEC)	Motor vehicle collision + mld Traumatic Brain Injury	2 wks	Superior parietal gyrus (thicker in TEC compared to mTBI)	Superior parietal gyrus, medial orbiofrontal gyrus, lateral orbitofrontal gyri (more active in TECs without mTBI). SPG activity (greater in resiience)	
Wang et al. (46)	38	16 PTSD, 22 TEC	62, 73	31.6 ± 9.5 (PTSD), 34.7 ± 13.2 (TEC)	Motor vehicle collision	2 wks + 3 mo	Left superior frontal gyrus volume (less decline in resilience)	dmPFC, vmPFC, insular cortex activity (decreased in resilience)	x
Busso et al. [73]	51	11 abused, 33 control	60.8	16.96 ± 1.51 (13–20)	Childhood maltreatment	Varied	Cortical thickness: prefrontal lobe, temporal lobe (decreased in trauma-exposed); m temporal gyrus & parahippocampal gyrus (decreased in resilience)	x	x
Gilam et al. [26]	46	29 soldiers, 17 civilians	0	19.86 ± 1.06 (soldiers), 19.24 ± 0.44 (civilians)	Military training	pre-trauma + 1 yr	x	vmPFC, locus coeruleus (increased in resilience)	x
Harms et al. [113]	54	29 early life stress, 25 low stress	52	11.2 (9–13)	Early life stress	Varied	x	dlPFC activity (greater in resilience)	x
Lin et al. [18]	50	All stress exposed	0	18.86	Military training	Pre-trauma	x	Amygdala electrical fingerprint (decreased in resilient)	x
Mangelsdorf [50]	22	childbirth related stress	100	28.1 ± 3.15	Childbirth	1 mo + 4 mo	vmPFC grey matter (higher persoal growth initiative pre-trauma predicted larger volume)	x	x
Stevens et al. [42]	31	All TE	48	31.9 ± 10.4	Mixed	1 mo + 3 mon + 6 mo + 12 mo	x	Amygdala (decreased in resilient), ventral ACC (increased in resilient)	x
Terpstra et al. [55]	80	All had moderate to severe TBI	28	39.4 (17–80)	Traumatic Brain Injury	5 mo, 12 mo, 30 mo	Hippocampus (increased in resilient)	x	x
Whittle et al. [110]	166	varying	49	Ages at each time point: 12.79 ± 0.43, 16.70 ± 0.52, 19.08 ± 0.46	Childhood maltreatment	Varied + 4 yrs + 7 yrs	Hippocampus: CA4-DG (increased development in maltreated youth), presubiculum, CA1 (increased development associated with early- and late-onset psychopathology)	x	x
Hu et al. [44]	70	29 PTSD, 41 TEC	55	37.2 (18–60)	Motor vehicle collision	2 days	frontal-temporal cortex, left insula, left rACC (increased in resilient)	x	x
Meng et al. [90]	22	All TE	36	38.4	Natural disaster	25 days + 2 yrs	Posterior limb of internal capsule, superior and posterior corona radiata (SCR and PCR), and external capsule FA (increased in TEC), superior corona radiata FA (increased in resilent).	x	x
Quidé et al. [54]	45	10 PTSD, 15 TEC, 20 HC	100	24.2 (18–53)	Sexual assault	3 wks	Hippocampus (increased in resilient)	x	x
Saxbe et al. [109]	22	Healthy but varied degrees of trauma exposure	43	12.99	Community violence	3–5 yr	Hippocampus, Amygdala (increased in resilient)	x	Hippocampus-frontotemperal lobe (decreased in resilient)
van Rooij et al. [57]	27, 31	All TE	48, 35	31.5, 36.9	Mixed	1–2 mo	x	Hippocampus (higher in resilient)	x
White et al. [45]	21	All combat exposed	19	30.64 (21–44.8)	Military deployment	2 mo + 6–12 mo	x	dACC, inferior frontal gyrus/anterior insula, inferior parietal cortex (increased in resilient)	x
Xie et al. [52]	44	All TE	70	32.8 (19–58)	Motor vehicle collision	2 wks + 3 mo	Hippocampus (increased in resilient)	x	x
Yoon et al. [61]	59	30 PTSD, 29 HC	63	26.7	Fire	1.4 yrs + 2.7 yrs + 3.9 yrs	x	x	The amygdala–insula & amygdala–PFC (strengthened, then normalized), amygdala–thalamus (normalized during recovery), amygdala–hippocampus (low across timepoints), amygdala–PFC connectivity (greater in resilient)
Zsoldos et al. [76]	349	All healthy	19	69.6	Allostatic load	Varied	Grey matter density (increased in resilient)	x	x
Ben-Zion et al. [53]	171	All TE	50.8	34.2 (18–65)	Mixed	1 mo + 6 mo + 14 mo	Hippocampus (increased in resilient), cavum septum pellucidum (decreased in resilient)	x	x
Heyn et al. [74]	48	27 PTSD, 21 HC	Not listed	13.92 ± 2.44 (PTSD), 14.01 ± 2.81 (HC)	Mixed	Varied + 1 yr	Right vmPFC and bilateral vlPFC (increased in resilience), dlPFC (decreased in resilience)	x	vmPFC-amygdala, vlPFC-hippocampus (increased in resilience)
Heyn [77]	55	10 PTSD remitter, 18 PTSD nonremitter, 27 HC	69	13.28 ± 3.45 (PTSD Remitter), 14.21 ± 2.46 (PTSD nonremitter), 14.16 ± 2.70 (HC)	Mixed	Varied + 1 yr	vlPFC surface area (decreased in resilience), frontal pole surface area, vmPFC thickness (increased in resilience)	x	x
Cwik et al. [21]	56	21 ASD, 17 PTSD, 18 HC	57	34.76 ± 12.62 (ASD), 37.35 ± 15.56 (PTSD), 30.11 ± 12.14 (HC)	Mixed	2 wks + 1 mo	Visual cortex, occipital, PFC (increased in resilient); middle temporal gyrus/superior temporal gyrus (reduced in resilient), amygdala and hippocampus (no significance)	x	x
Webb et al. [65]	48	All TE	72	33.4	Motor vehicle collision, physical assault, mixed	2 wks	x	x	PAG-frontal pole, PAG-posterior cingulate cortex (decreased in resilience)
Belleau et al. [121]	54	14 PTSD, 40 TEC	65	33.22 (11.55)	Mixed	2 wks	x	x	Amygdala-cerebellum and amygdala-postcentral gyrus fsFC (increased in resilient), amygdala-postcentral gyrus and amygdala-midcingulate cortex (increased in resilient during trauma recall)
Fani et al. [122]	30	31 Trauma exposed, 21 w/o Posttraumatic anedonia (PTA), 10 w/	67	33.1 ± 12.5 (no PTA), 32.9 ± 13.1 (PTA)	Mixed - ED	~1 mo	UF tract integrity (greater in resilient)	x	x
Koch et al. [31]	210	Healthy police officers at high risk for trauma exposure	27	24.02 ± 5.19 (18–45)	Police training	Baseline (pre) + 16 mo	Hippocampus (larger in resilient) and Amygdala (larger with more trauma exposure)	x	x
Quidé et al. [82]	44	10 PTSD, 15 TEC, 19 control	100	23 (18–53)(control), 25 (18–52)(victims)	Sexual assault	3 wks + 6 mo	x	x	Right middle/superior occicpital gyrus (lower centrality in resilience), PCC/precuneus (reduced centrality in TEC compared to HC)
Harnett et al. [80]	109	109 TE	70	35.31 ± 12.97	mixed	2 wks	x	x	dlPFC-threat areas (increased in resilience), inferior temporal gyrus-DMN (decreased in resilience.
Kaldewaij et al. [41]	185	All TE	24	23 (18-45)	Mixed - Police Work	Baseline + ~16 mo	x	anterior PFC, dorsal and medial frontal (increased in resilience), AMYG (predicted trauma exposure)	x
Grueschow et al. [25]	48	All TE	58	24 ± 1.99	Medical Residents in ED	Baseline (pre-internship)	x	Locuscoeruleus (increased in resilience)	Locus coeruleus - AMYG (increased in resilience)
van Rooij et al. [58]	28	All TE	35.7	29.36 ± 12.46	Mixed	2 mo	x	hippocampus (increased in resilience)	x
Weis et al. [56]	208	All TE	55	33.1 ± 10.8	Injury	2 wks + 6 mo	hippocampal subfield volumes (no significance)	x	x
Harnett et al. [48]	75	All TE	40	35.24 ± 12.53	Mixed – primarily auto accidents	1 mo + 12 mo	UF and Fornix FA (higher in resilient). vmPFC and precuneus grey matter volume (increased in resilient)	x	x
Stein et al. [43]	421	All TE with TBI	33.5	38.7 ± 16.08	Traumatic Brain Injury	2 wks	superior frontal, rostral, and caudal ACC (larger in resilience). Principal component analysis using sfACC, rACC, cACC, and insula (predicted resilience)	x	x

We discuss the findings following general principles outlined by Williams et al. [8], in terms of the role of each region within a larger established network. Emerging findings suggest that network-level alterations provide a parsimonious explanation of psychiatric symptoms [9, 10]. For example, neuromodulation therapies for depression, obsessive-compulsive disorder, and Parkinson’s disease show similar symptom-reducing effects across multiple nodes within a target network [11, 12]. Such findings indicate that the network may be the ideal unit of analysis for treatment targeting and characterization of psychiatric disorders. We also theorize that summarizing findings across networks will allow patterns to emerge which may not be clear at the single-region level. We, therefore, discuss the role of regions typically engaged within the default mode, salience, threat, reward, attention, and cognitive control networks, as well as primary and associative sensory cortices. This is further elaborated in Box 1.

Box 1 Canonical neural circuits contributing to post-trauma resilience We organize our discussion of neural features contributing to resilience around an established framework relating neural circuits to psychopathology [8]. We also integrated information from Cai et al. [123], and Yeo et al. [124] to ensure overlap with task-based and resting-state network parcellation approaches. Although well-established findings indicate that brain regions can participate in multiple networks depending on task-related and internal demands, here we organize regional findings into canonical networks of regions that are tightly coupled at rest and during particular types of tasks, as follows:

*Threat and Salience:* amygdala, hippocampus, insula, and both dorsal and ventral portions of the prefrontal cortex, including the dorsal medial prefrontal cortex (dmPFC), dorsal anterior cingulate cortex (dACC), and the ventral mPFC (vmPFC) and rostral ACC (rACC)

*Reward:* ventral striatum and projections to the orbitofrontal cortex (OFC) and vmPFC

*Default mode:* mPFC, angular gyrus, posterior cingulate/precuneus, medial temporal lobe, and ventrolateral PFC (vlPFC)

*Attention:* Medial superior frontal cortices (msPFC), aI (auditory), superior/inferior posterior parietal cortices

*Cognitive control:* dorsolateral prefrontal cortex (dlPFC), ACC, precentral gyrus (PCG), dorsal parietal cortex (DPC)

## Pre-trauma predictors of resilience

Longitudinal studies have proved to be a powerful tool in identifying aspects of neural structure and function that contribute to resilience. One particularly important goal has been to search for traits that exist prior to a trauma, which confer protection from chronic stress-related symptoms following the trauma. Due to challenges in predicting who will be exposed to trauma and when, it has been difficult to study pre-existing neural traits of resilience. However, an emerging literature across both military-related and civilian longitudinal studies has captured pre-trauma structural and functional neural features that predict changes in later brain function and adaptive natural recovery from stress-related symptoms after trauma. These studies provide key insights into neural resilience and how the brain changes and recovers after exposure to psychological stress.

### Threat and salience

The majority of research on risk and resilience factors for trauma-related psychopathology links the psychological response to trauma with individual differences in the structure and function of areas associated with threat processing, which has some overlap with the salience network. The areas discussed can be categorized by those involved in threat inhibition (hippocampus, vmPFC), versus those involved in threat response and salience detection (amygdala, insula, dACC, dmPFC).

#### Pre-trauma protective factors

The amygdala is widely studied in trauma research due to its role in responding to threatening stimuli and the expression of fear (for review, see ref. [13]). It has been hypothesized that individual differences in the structure and function of the amygdala may contribute to stress resilience, such that individuals who show less threat reactivity may be more resilient after trauma [14]. There is now evidence to support this idea across a variety of different cohorts, in which individuals with less amygdala reactivity measured before trauma exposure were more resilient and reported fewer PTSD symptoms post-exposure, in children studied prior to a terrorist event [15] and young adults prior to military deployment [16–18]. These effects appear to generalize beyond PTSD to more general stress-related symptoms. For example, healthy young adults who showed less amygdala reactivity to emotional faces at an initial study visit went on to report fewer stress symptoms following stressful life events that occurred 3 months to 4 years later [19]. Lower amygdala reactivity to face stimuli has also been linked with fewer later depressive symptoms [20]. However, individual differences in pre-trauma reactivity may not be accompanied by gross structural differences—van Wingen and colleagues found that amygdala volume pre-trauma does not appear to be predictive of resilience post-trauma [21–23].

Individuals with less amygdala reactivity may also show less sympathetic output when new threats are encountered. For example, among medical students scanned prior to a stressful ED internship, less connectivity between the amygdala and a key downstream noradrenergic output region the locus coeruleus (LC) [24] measured before the internship predicted later resilience to anxiety and depression symptoms, as did lower reactivity of the LC during an emotional conflict task [25]. These studies show that pre-existing differences in amygdala and its regulation of sympathetic nervous system activity may predict resilience to stress in some individuals. Resilient individuals may be better able to modulate the activity of the amygdala in concordance with current environmental demands.

Similarly, the ventromedial prefrontal cortex (vmPFC) and its connections with the amygdala or hippocampus may be important contributors to adaptive threat responses. The vmPFC implements top-down control over fear-related regions like the amygdala, and helps to adaptively modulate threat responses [13]. A recent study found that greater vmPFC activation pre-combat training positively predicted resilience to stress symptoms post-training [26]. Structural studies of earthquake survivors who were scanned prior to the event have found that greater grey matter volume (GMV) in the right rACC [27] predicted less anxiety and PTSD symptoms measured 3–4 months after the event. In the same cohort of earthquake survivors, Sekiguchi and colleagues also found that higher pre-trauma fractional anisotropy (FA) of the right anterior cingulum, a tract that is part of the larger cingulum bundle connecting several PFC and temporal lobe regions, was predictive of fewer anxiety and PTSD symptoms evaluated 3–4 months after trauma exposure [28]. Together, these findings link greater vmPFC function, volume, and connectivity with stress resilience.

The hippocampus is another region likely to aid in appropriate regulation of physiological arousal responses to threat, known to be important for contextualizing memories and experiences [29]. Classic findings implicating the hippocampus in resilience came from twin studies of combat veterans and their twins who were not exposed to combat stress. Veterans who were diagnosed with PTSD and their unexposed twins had lower hippocampal volumes compared to veterans with similar exposures but without PTSD and their twins [30]. This suggested that greater hippocampal volume may be a familial PTSD resilience factor. However, this work was conducted many years after the trauma had occurred. A more recent study provided confirmatory observation of hippocampal volume prior to the stressful training experiences. In police recruits, Koch et al. found that greater left hippocampal dentate gyrus volume measured before training predicted fewer PTSD and stress symptoms 16 months post-training [31]. Similarly, in functional studies with a civilian cohort exposed to a terrorist attack, greater bilateral hippocampal activity during an emotional regulation task pre-exposure to trauma was predictive of resilience to PTSD symptoms measured post-exposure [15], suggesting another potential inherent resilience trait. However, another study of military recruits reported no significant difference in hippocampal response to stressful stimuli measured before trauma exposure and the association with post-exposure symptoms [16]. This discrepancy in pre-trauma functional results may be explained by the context in which their traumas occurred and the mental preparedness of the military cohort fulfilling their service versus the civilian cohort. Overall, evidence suggests that greater hippocampal volume before a trauma may be protective, but future longitudinal studies will be needed to determine if pre-trauma function relates to resilience after trauma exposure and how context of the trauma may impact these results.

The salience network has been implicated in the monitoring of affective environmental cues. Structural and functional differences in distinct subregions of the ACC may denote specific PTSD resilience factors. Studies of combat exposed veterans and their unexposed twins have found that less resting state dACC and mid-cingulate activity [32], as well as less responsivity of the dACC during a cognitive interference task [33] were associated with resilience to the development of PTSD. Less functional activity in the dACC pre-trauma may therefore be a protective factor. These studies indicate that less dACC activity and greater ACC volume or connectivity via the anterior cingulum may be protective traits.

#### Pre- to post-trauma changes

Regardless of symptom presentation, regions involved in threat detection and response, including the amygdala and insula [22, 31, 34] have generally been found to increase in volume and threat reactivity from pre- to post-trauma exposure. Changes in these regions may be dependent on the actual or perceived severity of the trauma experienced. Participant reports of perceived threat during military deployment were correlated with pre- to post-deployment increases in amygdala-insula and decreases in amygdala–dACC functional connectivity (FC) in response to social threat cues 1.5 months after deployment [22]. However, data collected in the same military cohort 1 year later showed that amygdala activity, insula activity, and amygdala-insula FC returned to pre-deployment baseline levels, while only decreased amygdala–dACC coupling remained significantly correlated with perceived stress [23]. Therefore, changes in amygdala and insula as a result of trauma may not necessarily be permanent.

A pattern of conserved hippocampal volume and function from pre- to post-exposure to stress has also been associated with resilience. Hippocampal volume appears to decrease as a function of stress exposure; for example, individuals who experienced a greater number of stressful life events demonstrated larger decreases in left parahippocampal and right hippocampal volumes [35]. Thus, it seems that inherently larger hippocampal volume, which is then sustained post-trauma, may indicate individuals who are less at risk for developing PTSD. However, the individual’s response to the stressful event appears important—soldiers who showed less change in hippocampus reactivity to threat stimuli from pre- to post- deployment reported lower stress symptom severity post-deployment [16]. In this same study, increases in hippocampal-vmPFC functional coupling from pre- to post-deployment predicted resilience, as measured by fewer stress-related symptoms [16]. Therefore, while larger hippocampal volume is protective, individuals with less hippocampal reactivity but improved connectivity from pre- to post-trauma may be more resilient.

The uncinate fasciculus (Uf) is the primary white matter tract connecting the vmPFC and orbitofrontal cortex to the amygdala, and is involved in emotional processing [36]. Standard neurocognitive models of stress resilience predict that greater integrity of the Uf should facilitate regulation of the amygdala by the vmPFC, enhancing resilience. However, detailed investigation of this tract among earthquake survivors suggests that there is a more complex temporal association between Uf integrity and resilience that unfolds over the course of recovery. Sekiguchi and colleagues found that a longitudinal increase in FA of the left Uf from pre- to 3+ months post-trauma was negatively associated with resilience, measured by more anxiety symptoms post-trauma [28]. However, by the time of a 1 year follow up, participants who had initially shown the increase in Uf FA now showed a decrease in both left and right Uf FA, indicating recovery of this tract even if symptoms did not significantly improve [37]. Although pre-trauma results suggested that greater anterior cingulum bundle (Cg) FA is a protective factor, additional analyses of these same earthquake survivors reveal that increased left Cg FA from pre- to 3+ months post-exposure was associated with higher anxiety scores and therefore less resilience [28]. Likewise, PTSD severity was positively correlated with increased FA of the right Cg 1+ year later [37]. These studies imply that individuals who were resilient, showing fewer chronic PTSD or anxiety symptoms in the aftermath of the earthquake, were less likely to show any changes in anterior cingulum white matter diffusivity from pre- to post-exposure. Additionally, changes in GMV of the bilateral ACC were negatively correlated with the number of stressful life events experienced within a 3 month time period [35], suggesting that exposure to stressful experiences could lead to reductions in ACC volume, though this was only demonstrated in one small study. It is possible that more resilient participants with initially greater anterior cingulum FA and ACC volume did not experience as great of changes from pre- to post-exposure due to a ceiling effect. However, similar to results with Uf FA, over the course of a year, participants that initially had increased r Cg FA showed some reductions and recovery of the tract [37]. These studies indicate that greater changes of the anterior cingulum bundle are associated with less resilience to trauma but may not be permanent changes to the brain.

### Reward

Very little work has focused on the reward circuit pre- to post- trauma exposure. One study found that differences in nucleus accumbens (NAc) activation pre-deployment did not predict resilience, but that at the post-deployment assessment, more resilient individuals showed greater NAc response to reward compared to individuals experiencing worse post-trauma PTSD symptoms [17]. Additionally, studies of earthquake survivors found that individuals with fewer PTSD symptoms 3 months post-trauma showed less of a decrease in GMV of the left orbitofrontal cortex (OFC) from pre- to post-trauma [27]. Based on these studies, it appears that pre-exposure differences in reward circuitry may not indicate any protective factors, but that resilient individuals retain higher reward circuitry response and structure after trauma exposure. As blunted affect is a symptom of both PTSD and depression, it is imperative that future longitudinal studies further explore the relationship between trauma exposure and changes in reward circuitry.

### Cognitive control

The anterior prefrontal cortex (aPFC) and in particular the frontopolar cortex is thought to be involved in action selection and reasoning [38, 39]. Koch and colleagues further suggest that the aPFC and lateral frontal pole may be important for emotional cognitive control and switching between alternative emotional strategies during cognitive decision-making tasks [40]. A recent study of a large cohort of police recruits found that greater pre-trauma anterior PFC, dorsal and medial frontal pole response during an approach-avoidance task was associated with fewer PTSD symptoms measured after exposure to trauma [41]. This suggests that having greater top-down emotional control may be another protective trait against the development of PTSD. Perhaps more resilient individuals with greater aPFC activity are better able to adaptively alternate their emotional response in the face of trauma.

### Summary of pre-trauma resilience factors

**Fig. 1:**
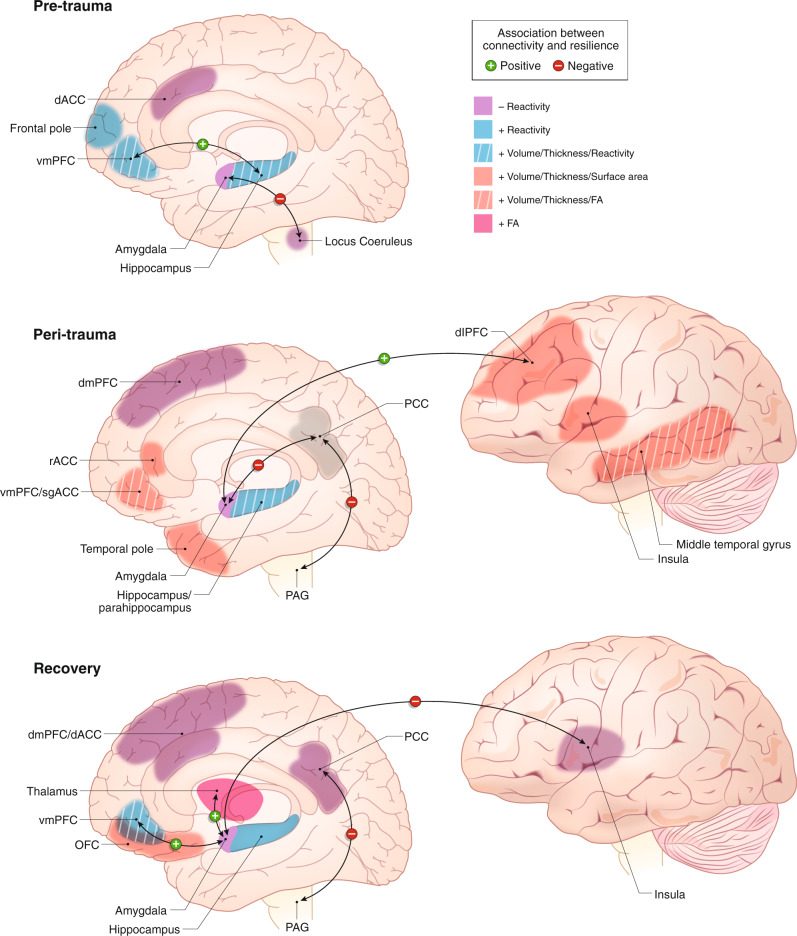
Neural model of resilience to trauma—pre-trauma factors, peri-trauma predictors of resilience, and recovery factors. *Pre-trauma* factors of resilience include larger vmPFC and hippocampus volume, greater activation of emotional regulatory regions like the vmPFC, hippocampus, and aPFC, and less activation in threat response regions such as the amygdala, dACC, and LC. Resilient individuals demonstrated fewer functional and structural changes from pre to post trauma compared to those with symptoms of PTSD. However, greater coupling between the vmPFC and hippocampus from pre to post trauma is associated with resilience. *Peri-trauma* features that predict later resilience include larger structural features in the hippocampus, parahippocampus, vmPFC, sgACC, dlPFC, temporal lobe, as well as greater rACC surface area volume. Similar to pre-trauma, decreased amygdala and increased hippocampal/parahippocampal functional activity soon after trauma are associated with resilience. Increased dlPFC-amygdala and decreased PCC-amygdala or PCC-PAG connectivity positively correlate with later resilience. Notably, however, many of these findings appear the opposite among those at risk for chronic PTSD with dissociative features. Over the course of *recovery*, functional reactivity in the amygdala, insula, and dACC decrease or return to pre-trauma levels. Amygdala-insula, -thalamus, and -vmPFC connectivity also return to baseline levels, and connectivity between the PCC and PAG continues to weaken over time. Structural increases in frontal regions such as the OFC or vmPFC as well as increases in the thalamus are related to recovery. There is greater activation of emotional regulatory regions like the vmPFC and hippocampus as well as less activity in the dmPFC and PCC. Yellow/blue fill = positive/negative correlations between resilience and functional activation or reactivity. Orange/blue border = positive/negative correlation between resilience and structural volume. Green/blue arrows = positive/negative correlation between resilience and connectivity between regions.

Potential resilience or protective factors prior to a trauma (Fig. 1) include lower engagement of threat response and salience detection regions, such as the amygdala, insula, LC, and dACC. Likewise, greater hippocampal volume, vmPFC, and anterior prefrontal activation pre-trauma may protect against later effects of trauma. Individuals with greater resilience demonstrate regional conservation of volume, function, and connectivity from pre- to post-trauma across multiple networks, as well as increased hippocampal-vmPFC coupling. However, pre-trauma studies have not yet reported findings in networks including dorsal and ventral attention, default mode, or sensory cortex. Future research evaluating brain-wide, multi-circuit interactions in individuals before and after trauma exposure is necessary to determine the potential involvement of other networks in predicting pre-trauma resilience.

It is important to note that while being a “resilient” individual with a dampened threat and salience response or greater threat inhibition may be adaptive in times of safety and security, these same traits may be harmful in situations where responding to threat cues is vital for survival, like in the case of a soldier on the battlefield. Trait-like, lower reactivity to threatening cues may not keep a person safe in times of real danger. Resilience may therefore be reflected in the adaptive regulation of circuits that modulate autonomic and emotional arousal, but not defined by any specific level of regional reactivity.

## Peri-trauma to long-term predictors of resilience

Most civilian trauma neuroimaging research occurs in the aftermath of trauma, during peri-trauma and in the years following. The main focus of this research has been to establish structural and functional differences or changes that predict the future emergence of chronic trauma-related symptoms or resilience. As many trauma-exposed individuals seek acute treatment for injuries or related medical issues, the peri-traumatic period is a key time window for early assessment of stress resilience and for the deployment of early interventions to boost resilience and recovery.

### Threat and salience

The majority of peri- and post-trauma neuroimaging findings implicate the structure and function of areas involved in threat inhibition (hippocampus, vmPFC) and those involved in threat response and salience detection (amygdala, insula, dACC, dmPFC).

Consistent with pre-trauma findings, amygdala reactivity to threat cues acutely post-trauma correlates negatively with later resilience. For example, in participants recruited from the emergency department (ED), who were scanned 1-month post-trauma, amygdala reactivity to negative emotional stimuli negatively correlated with resilience, as measured by fewer PTSD symptoms months later [42]. While this occurs in the likely context of a pre- to post-trauma increase in amygdala reactivity [22, 34], relatively lower post-trauma amygdala reactivity may indicate PTSD resilience. However, the relationship with amygdala structure remains unclear. Stein and colleagues showed no association between amygdala volume 2 weeks following a head injury trauma and later PTSD symptoms [43], although there have been few early posttrauma studies of amygdala volume with respect to later symptoms or well-being.

Other areas of overlap between the threat and salience network include the dACC and insula. Within these areas, increased reactivity and structural integrity are associated with resilience. A pattern of larger insula, dACC, and rACC volume assessed shortly after trauma predicts fewer future PTSD symptoms after head injury trauma [43], and motor vehicle accident [44]. Greater structural integrity in this network may lead to adaptive regulation of emotional responses to threat stimuli. However, functional imaging findings have been more mixed. PTSD symptom improvement in military service members over the first few months following the return from deployment was positively correlated with engagement of the anterior insula and dACC during an affective task requiring effortful attention allocation in the face of distractors [45]. In contrast, PTSD symptom improvement was negatively correlated with dmPFC engagement during a less effortful emotional appraisal task 2 weeks after motor vehicle collision and with insular engagement 3 months after. This study also showed an increase in dmPFC activation from 2 weeks to 3 months which was accompanied by an increase in PTSD severity over this time [46]. This network’s contributions to trauma recovery may therefore depend on its role in various task demands, such as attention allocation, and depend on emotional context and nature of the trauma. Further work is needed to disentangle the relative contributions of these effects, employing tasks with varying effort levels or attentional complexity, and comparing across trauma types.

The amygdala is regulated by the vmPFC, and longitudinal findings support that an increase of vmPFC morphometry or activity is important for later resilience. Among PTSD patients scanned at varying times since trauma, a longitudinal increase in sgACC over 6–9 months predicted natural recovery over the same timespan [47]. Similarly, trauma survivors with greater rACC and vmPFC surface area and volume collected peri-trauma were less likely to develop PTSD symptoms three to twelve months later [43, 44, 48]. DTI studies also implicate the vmPFC in resilience, finding larger FA and less mean diffusivity in the vmPFC predicted resilience 2 days following a traumatic event, and larger vmPFC FA predicted lower PTSD severity at 1 or 6 months later [49]. Interestingly, a study of prenatal mothers found that greater pre-delivery personal growth initiative correlated with larger maternal vmPFC grey matter volume after birth, indicating a link between proactive coping and vmPFC structure post-trauma [50]. Concerning functional findings, activation to trauma-related pictures in bilateral vmPFC 2 months post-trauma positively correlated with resilience to acute PTSD [51]. Similarly, persistent activation of the sgACC over repeated threat stimuli presentation was positively associated with PTSD resilience months later [42]. Although there are some mixed findings [46], overall, greater vmPFC volume, FA, and engagement in the context of threat predict less PTSD severity post-trauma, consistent with the role this area plays in inhibiting fear responses.

Hippocampal activation and structural integrity overall appear to be predictive of resilience. Studies of hippocampal structure suggest that greater hippocampal volume correlates with greater resilience to trauma, with larger hippocampal volumes early post-trauma among those who demonstrated the fewest future PTSD symptoms [52–54]. However, studies also suggest that the experience of stressful life events correlates with smaller hippocampal volumes weeks and months post trauma, even among resilient individuals who demonstrated few PTSD symptoms after trauma [35, 54]. This may be compounded among individuals who continue to experience high levels of distress following the trauma, for example, high anxiety symptoms 5–12 months following traumatic brain injury (TBI) predicted atrophy of the right hippocampus between 12 and 30 months post-trauma [55]. When individual hippocampal subfield volumes were assessed in individuals two weeks and six months post-trauma, no correlation with PTSD symptoms was found [56], although this study was underpowered to assess group differences in PTSD and had low PTSD severity as a whole. These structural differences appear to be accompanied by differences in function. Greater engagement of the hippocampus during response inhibition 1-month post-trauma predicts PTSD resilience, with individuals who experienced similar types of traumas showing lower symptom severity up to 6 months post-trauma [57]. Hippocampal activation during fear conditioning also positively correlates with trait resilience at two months post-trauma, as measured by the Connor-Davidson Resilience Scale (CD-RISC) [58]. Furthermore, longitudinal increases in hippocampal activity in response to social threat cues and during fear extinction are associated with PTSD symptom improvement several months later [58, 59]. It may be that traumatic events result in smaller hippocampal volume and impaired function, or that smaller hippocampal volume or reduced function is a vulnerability factor. However, these effects may be seen to a lesser degree in resilient individuals who show fewer symptoms of anxiety and PTSD. Larger hippocampal volumes and greater engagement in inhibition and extinction, in turn, appears to facilitate later reductions in PTSD symptoms in the post-trauma recovery period, and correlate with trait resilience. It is thought that hippocampal activation during fear conditioning tasks may lead to better contextual threat processing, which then results in improved contextual behavior modulation [58].

Connectivity within and to the threat network appears highly important to the response to the trauma, with many studies finding amygdala FC to be involved in fear learning and symptom severity following trauma. Amygdala-PFC, -PCC, -dACC, -superior temporal gyrus, and -insula functional connectivity have all been implicated in recovery from trauma-related psychopathology. Amygdala-PFC connectivity, and PFC sub areas, has previously been supported as a resilience factor [60]. The vmPFC is suggested to inhibit fear responses via the amygdala [13]. Concurrently, DTI studies point to greater amygdala-PFC or Uf structural connectivity as a protective factor for resilience, as measured by fewer PTSD symptoms [61]. Increased post-trauma connectivity negatively associated with longitudinal resilience include amygdala-PCC [62], -dACC [23], -left superiortemporal gyrus [63], and with somatosensory areas [64]. As these areas are part of the default mode network and sensory network, higher connectivity in PTSD between these areas may reflect hypervigilance, increased emotional self-reflection, or hypersensitivity to sensory signals. Increased PAG connectivity, which receives output from the amygdala and hippocampus, with the PCC and frontal pole is also shown to negatively correlate with resilience 6 months posttrauma [65]. One study, which monitored shifts in amygdala connections in PTSD patients post-trauma for 5 years using DTI, found that amygdala-insula connectivity initially strengthened and then normalized during recovery from PTSD symptoms, -prefrontal cortex connectivity initially was unchanged, strengthened, and then normalized, -thalamus connectivity normalized during recovery, and -hippocampus connectivity remained low [61]. This highlights the potential complexity in network dynamics during recovery from PTSD, and the utility of assessment over several years.

These findings support proposed models suggesting that hippocampus and vmPFC modulation of amygdala activity early post-trauma supports resilience and recovery. On a basic level, less amygdala reactivity, greater hippocampus, and vmPFC activity and volume, and greater functional and structural connectivity between these regions and the amygdala early post-trauma correlate with better PTSD recovery. However, evidence across the collected studies suggests that functional correlations are task dependent, with vmPFC engagement across repeated presentations of threat correlating with quicker recovery, whereas the vmPFC response to threatening versus neutral social cues was associated with greater future symptom maintenance. Similarly, in the hippocampus, engagement during inhibition is associated with less future PTSD symptom symptoms, but greater response to threat cues during conditioning correlates only with trait resilience. Therefore, a multifaceted approach is necessary to fully understand these three areas of the threat network. Methods for assessing resilience more broadly than PTSD symptoms and recovery are necessary.

### Reward

In the peri-trauma period, again, a very limited set of studies have focused on reward circuits and resilience. One longitudinal study in cancer survivors did find that GMV in the right orbitofrontal cortex 3–15 months post-surgery and two years later was significantly larger in resilient survivors and non-trauma controls compared to those with PTSD [66], which in line with findings from a pre- to post-trauma study [27]. Orbitofrontal cortex activation correlates positively with magnitude of presented reward [67] and reward “pleasantness” [68], and dysregulation of this area may play a part in anhedonia symptoms post-trauma. However, reward circuits are also highly overlapping with areas involved in the threat network, including the amygdala and mPFC regions [8], and thus may also play a role in emotional regulation [69, 70]. Considering that a decrease in positive affect is a symptom of PTSD, further research understanding the relationship between resilience and reward circuits is necessary.

### Default mode

Default mode network (DMN) activation is observed while at rest and during spontaneous reflection [8, 71, 72]. The role of the DMN in resilience is highly consistent across early post-trauma studies. Structurally, greater FA across nodes of the network [49], greater cortical thickness [73], GMV [21, 44,74–76], and surface area [77] within DMN nodes all positively predict resilience weeks and months following trauma. This may extend from the core DMN to the temporal subnetwork as well, as one study showed that individuals with increasing PTSD symptoms over time also demonstrate accelerated temporal lobe atrophy [78]. While these findings suggest that structural integrity of the DMN post-trauma promotes resilience, functional studies found a negative correlation between PCC reactivity and DMN-threat network connectivity and early resilience. Early post-trauma, the PCC response to trauma-related images correlates negatively with resilience [79]. Acute post-trauma connections between DMN nodes and aspects of the threat network appear to additionally reduce resilience, and may be particularly related to re-experiencing symptoms. Post-trauma DMN-amygdala FC predicts PTSD severity at time of scan, and negatively correlates with resilience months later [62]. Early post-trauma connectivity between DMN nodes and rACC, periaqueductal grey, inferior frontal gyrus, and thalamus also are all negatively associated with subsequent resilience [62, 65, 80, 81]. Further, reduced centrality of the PCC/precuneus in sexual assault victims peri-trauma is reported in those who did not develop PTSD, compared to non-trauma exposed controls [82]. Thus, early post-trauma DMN engagement via the PCC and threat network connectivity is suggested to have a negative impact on resilience, mainly within the first year following trauma.

While DMN engagement and threat-network connectivity predict acute trauma-related psychopathology, they may lead to later recovery and long-term resilience. For example, reactivity in the DMN to trauma-related pictures two months post-trauma was negatively correlated with early PTSD resilience, but positively related to resilience 2 years later [51]. Participants in this study showed a general reduction in PTSD symptoms over the two years, and a reduction of PCC and mPFC activation between time points, suggesting that early DMN activation which then decreased within the months following trauma was eventually an indicator of adaptive recovery. Such findings illustrate the major value of longitudinal studies in uncovering unexpected ways that resilience may unfold over time. Further work is necessary to understand how early activation of the PCC post-trauma may play a role in resilience, both peri-trauma, and in the years following, and how FC within and with the DMN may or may not fluctuate over time. These findings also suggest that the DMN may play an important role in treatment and recovery. There is some support for this, with DMN-dlPFC, -ACC, and salience network connectivity increasing pre- to post-treatment in treatment studies [83, 84].

### Attention

The attention network is involved in directing and maintaining perceptual resources as a function of task demands [8, 85]. Emerging findings suggest that early post-trauma engagement of this network protects against the development of later trauma-related psychopathology. First, inferior parietal lobule response may change over time following trauma. Resilient mining accident survivors show greater activation of the inferior parietal lobule early post-trauma in response to trauma-related imaged compared to those with PTSD [51], suggesting that resilient individuals may initially maintain engagement of attentional regions to trauma related cues. In turn, sequential increases in inferior parietal responses to emotional stimuli 2–12 months post trauma predicted PTSD symptom improvement over this time in combat deployed soldiers [45], again pointing to an adaptive role of this network in trauma recovery. Increases in the function of this circuit over the post-trauma recovery period appear to have protective effects that extend beyond PTSD, for example, an increase in thalamus-dorsal attention network connectivity from 6 week to four months post-trauma correlated with decreased pain and post-concussive symptoms among mTBI patients [81]. Structural findings also support this possibility. Among soldiers deployed to Iraq/Afghanistan, larger volume and greater FA of the superior longitudinal fasciculus (a major tract connecting the parietal cortex with medial frontal regions) early posttrauma appeared protective against the later development of PTSD after military combat [63] and MVC [86]. Additionally, MVC survivors who developed PTSD showed a decrease in the volume of the left superior frontal gyrus from two weeks to three months post-trauma, whereas resilient participants did not [46]. Given that these findings span multiple types of traumatic experiences and different cohorts, this network may be an important and generalizable target for facilitating recovery.

### Cognitive control

The cognitive control network is involved in working memory and selective attention [8, 87, 88]. Within this network, changes in the structure and function of the dorsolateral prefrontal cortex (dlPFC) particularly appear to predict resilience. Adults showed greater dlPFC thickness over a year post-trauma exposure, and this thickness predicted greater recovery from PTSD symptoms over a period of 5 years [89]. These patterns suggest that growth of the dlPFC early after a traumatic event may be a positive adaptation. The dlPFC may engage in the regulation of emotional arousal responses during the early recovery period, as its connectivity with a threat network overlapping the bilateral amygdala and brainstem 2 weeks post-trauma predicts subsequent resilience to PTSD and depressive symptoms months later [74, 80]. This suggests the DLPFC as an interesting target for early intervention, following current neuromodulation treatments for chronic PTSD and MDD which typically target the dlPFC.

Alterations in the cognitive control network may also be related to its reciprocal projections with the thalamus. Traumatic events involving a head injury have been associated with decreased thalamus-frontoparietal control network FC [81], and FA in the thalamus and the superior corona radiata—connecting the thalamus and cortical areas—has been shown to significantly increase from within several months to years post-trauma exposure [37, 90], and this increase is associated with PTSD symptom recovery [90]. These adaptations to trauma may facilitate recovery as trait resilience scores positively correlate with FC between the inferior thalamus and middle frontal gyrus [91]. Cognitive control findings thus suggest that greater connectivity early post-trauma and increases in structural integrity from peri-trauma to years post-trauma promotes later resilience.

### Sensory

Sensory areas showing associations with longitudinal resilience include the occipital cortex and fusiform gyrus, involved in vision, and precentral cortex, involved in relaying and regulating sensory input. Vision related activation within the first year post-trauma appears to negatively correlate with resilience. Peritraumatic dissociation, a known risk factor for later chronic PTSD, depression, and chronic pain [92–94], positively correlates with activation along the right ventral visual pathway fusiform, lingual, and parahippocampal gyri during a trauma script task 2–4 months later [95], providing evidence for a negative correlation between visual circuit activation and resilience. Similarly, increased occipital centrality during resting-state three weeks following sexual assault predicted PTSD diagnosis six months post-trauma [82]. The occipital lobe is engaged during autobiographical recall of emotional events, and increased activation post-trauma may indicate a propensity for sensory reactivation during trauma-related, intrusive thought [82, 95].

### Summary of early and later post-trauma resilience factors

**Fig. 2:**
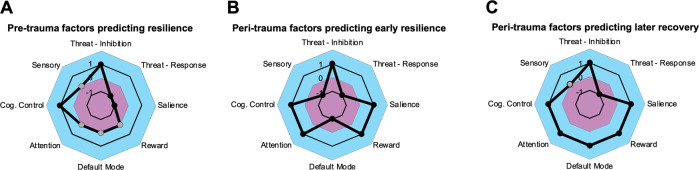
Network-level features predicting resilience, over time relative to trauma exposure. Across time following trauma, network patterns are noticeable. Pre-trauma findings (**A**) suggest that structural integrity/activation of inhibition areas in the threat and cognitive control networks positively correlate with resilience, while threat response and salience networks show negative associations with resilience. Post-trauma findings are split into two categories: early, peri-trauma findings that predict early resilience within the first year following trauma (**B**) and peri-trauma findings that predict later recovery and resilience years following the trauma (**C**). Correlations for the inhibitory and response areas of the threat network and cognitive control network are consistent over all three timepoints, and across the two post-trauma timepoints, the attention and reward networks both have positive correlations with resilience. However, some networks exhibit changes across time points. The DMN during peri-trauma negatively predicts early resilience, while it positively predicts later recovery and resilience. Changes are also seen across the salience network, with less salience activation pre-trauma predicting resilience, but higher salience activation post-trauma predicting peri-trauma resilience and later recovery. Axis values represent overall negative (purple) or overall positive (blue) correlations. Time points that do not have strong evidence supporting network correlations are indicated by grey dots.

Potential resilience protective factors post-trauma include increased vmPFC structure, reactivity, and connectivity, larger hippocampal volumes and activation, decreased amygdala reactivity, greater salience network activation and structure, and greater reward circuit/orbitofrontal cortex GMV (Fig. 1). A major risk factor appears to be amygdala reactivity and connectivity, which is highly involved in fear learning and symptom severity following trauma. Changes in amygdala connectivity during trauma recovery may be vital to resilience post-trauma. However, the resilient profile extends beyond the canonical circuits involved in threat detection, reward responsivity, and regulation of these functions. The pattern across networks is summarized in Fig. 2. Lower engagement of the DMN and DMN-threat network FC, and greater threat network-cognitive control network FC are also associated with resilience, particularly acutely, in the earlier months following trauma. Interestingly, while hyperactivation of DMN, hyperactivation of cognitive control network, and hypoactivation of the attention network are related to acute trauma-related psychopathology, findings suggest these features may be adaptations to the trauma which only have short-term consequences, and in fact predict longer lasting recovery in the years following trauma.

Peri-trauma and post-trauma studies to date are limited by the timing of scans, both in how early individuals are scanned post-trauma and later, longitudinal follow-ups. Neuroimaging studies face logistical constraints, in that most studies are unable to schedule individuals for a scan until weeks after a trauma occurs. In addition, most studies only evaluate trauma-related psychopathology or perform a second scan within the first year following trauma. This prevents evaluation of factors that may predict which individuals display long-term recovery and resilience compared to those with chronic trauma-related psychopathology. Studies that evaluate changes from peri-trauma to years post-trauma within these networks, and how they correlate with resilience over time, are necessary. Further, it is currently difficult to form conclusions regarding the influence of trauma type on findings, as many studies recruit individuals who have been exposed to all different kinds of trauma. While some studies are limited to motor vehicle collisions, a particular natural disaster, or military deployment, for example, the mix of time points that data is collected at and differences in neuroimaging data collected make it difficult to directly compare findings. Finally, while post-trauma longitudinal findings have been made within each of the described networks, the threat network and DMN have had the most findings, and the reward network the least. While the reported studies here reflect the importance of the threat network and DMN in particular in understanding resilience, they also elucidate gaps in the field regarding other network areas.

## Seeming contradictions in neurobiological resilience profiles

Several PTSD symptom clusters have a common neural correlate pattern. Specifically, intrusions, alterations in cognition and mood and altered arousal and reactivity symptoms are often associated with deficits in corticolimbic inhibition [96]. The findings outlined in the earlier sections suggest that greater engagement of cortical regions involved in emotion regulation before or shortly after a traumatic experience might then be protective against intrusion, mood, and arousal symptoms associated with posttraumatic stress. However, in a subset of individuals, this may not be the case. Fifteen to thirty percent of individuals with PTSD experience significant, chronic dissociative symptoms of depersonalization and derealization [97, 98]. These experiences of detachment from your sense of self, body and surroundings (e.g., “I feel like I’m looking at my body from outside my body” or “I feel like I’m looking at the world through a fog”) are associated with the opposite neural pattern to that described above. That is, these symptoms occur in conjunction with increased corticolimbic inhibition during emotionally provocative contexts [99]. Specifically, during symptom provocation paradigms, individuals with this subtype show a pattern of hypoactivity in the threat and salience networks in regions involved in salience detection (amygdala) and interoception (insula), and hyperactivity in cortical regions involved in threat inhibition, emotion, and bodily arousal regulation (e.g., vmPFC, dACC). Resting state FC supports this pattern with increased connectivity between subregions of the amygdala and the prefrontal cortex [100], the amygdala and insula [101], and the ventrolateral periaqueductal grey with cortical regions associated with passive responses to threat (e.g., temporoparietal junction) [102]. Furthermore, as discussed previously, peritraumatic dissociative symptoms of depersonalization/derealization are associated with subsequent activity in areas involved in vivid autobiographical memory recall (e.g., fusiform gyrus, lingual gyrus [95]). This suggests peritraumatic dissociation may play a role in the development of intrusive re-experiencing symptoms [95].

Given these findings, it follows that less vmPFC or dACC engagement before or shortly after a traumatic experience might be protective against developing both chronic dissociation and posttraumatic stress symptoms. To our knowledge, this has yet to be directly tested. However, a recent longitudinal treatment study in adolescents supports this conclusion. Specifically, this study demonstrated that improvements in dissociation post treatment were associated with decreased dACC and amygdala activation during an emotionally provocative task [103].

Taken together, some studies point toward more threat inhibition network activity and some point toward less as the profile of resilience. How do we begin to reconcile these seemingly opposite patterns of resilience? First, this evidence suggests there are different neural features at early timepoints that appear protective against different potential outcomes. More corticolimbic inhibition associated with threat inhibition and emotion regulation may protect against intrusions, alterations in mood and hyperarousal. However, too much corticolimbic inhibition may interfere with emotional learning [104, 105] and promote chronic dissociation and intrusive re-experiencing [95]. Second, it may be that each individual is on a continuum of possible over- vs. under-modulation in corticolimbic inhibition post-trauma (see the defense cascade model of dissociation [106, 107]). There may be a “goldilocks” level of just the right amount of inhibition that is associated with resilience toward intrusion, mood, hyperarousal symptoms and dissociative symptoms after trauma. Furthermore, this provides a contraindication for a one-size-fits-all approach to early interventions; different therapeutic approaches may be necessary depending on where an individual falls on this continuum.

## Special considerations related to development

Exposure to stress and traumatic events is thought to be particularly impactful during childhood, because of rapid brain changes that occur as part of brain maturation [108]. Neuroimaging studies on the longitudinal effects of trauma exposure suggest that early life stress exposure may be an important determinant of individual differences in the structure and function of key circuits involved in resilience. These early experiences may thus influence the way that these circuits adapt and respond in the face of new stressors or traumatic experiences.

Early stress and trauma seem to negatively impact threat and salience network structure and function. For example, early life stress predicted smaller bilateral hippocampal and amygdala volumes [109], with some suggestion of differences in the impact for boys versus girls [110], as well as sequential post-stress reductions in cortical thickness of medial and lateral prefrontal and temporal regions [73]. Also, more violence exposure was related to lower resting state FC between the right hippocampus and bilateral frontotemporal regions 3 to 5 years later [109]. Similarly, studies relating threat and salience brain findings to PTSD in children or adolescents demonstrate its importance for PTSD development versus recovery. The only pre-trauma prospective study showed that greater left amygdala reactivity and lower bilateral hippocampal activity in adolescents who were later exposed to a terrorist attack predicted development of PTSD symptoms [9]. Structural studies showed an association between PTSD severity and a reduction in hippocampal volume 12 to 18 months later was observed in maltreated children [111]. In a sample of maltreated girls but not boys, early onset of psychopathology was associated with the development of the right presubiculum, and late onset psychopathology with right CA1 volumes [42]. However, some studies report no significant difference in hippocampal volumes between those with or without PTSD across multiple timepoints, which could be due to sample size and younger age [112] or older age and lack of chronic PTSD [111]. A sustained right vmPFC GMV reduction and decreases in amygdala-PFC functional connectivity from baseline to a 1-year follow up were observed in children with PTSD, and volume reductions were found to be predictive of PTSD severity [74]. In terms of PTSD recovery, increases in vmPFC and sgACC thickness over time predict natural recovery from PTSD [73, 77]. These studies show profound effects of stress and different forms of trauma exposure reported during late childhood or early adolescence within regions of the threat and salience network that appear to influence later mental health following trauma exposure. Taken together, the findings in children are in line with the adult findings summarized in Fig. 2, with greater peri-trauma salience network structure and function positively predicting resilience or PTSD recovery.

While there is more developmental trauma research needed in networks other than threat and salience, a few findings have been published. For example, one observed that in a 10-year longitudinal study following children into adulthood, early stress exposure at the beginning of the study predicted less dlPFC engagement in error monitoring in adulthood [113], which is in line with observations among adults that more cognitive control is related to greater resilience. In contrast, a decrease in dlPFC GMV has been observed in sequential observations of youth with PTSD who remitted naturally, whereas those who maintained high PTSD symptoms showed dlPFC growth [74]. For the default mode network, reduced vlPFC-hippocampus connectivity is associated with PTSD diagnosis in adolescents [74]. This differs from adult studies of DMN connectivity, and may relate to unique effects of the temporal sub-circuit of the DMN with respect to the psychological adaptation to trauma, or because of the influence of developmental factors.

One important consideration for longitudinal studies in children or adolescents is the timing of traumatic events and its interaction with developmental period. The brain does not develop linearly, instead, there are specific sensitive periods for maturation of different cortical and subcortical structures [114]. The interaction with developmental periods significantly complicates the ability to determine the effect of a traumatic event on the brain, as this depends on the timing of the event and the stage of brain development, and both can vary widely within a study sample. Collecting measures of biological age as well as developmental (pubertal) stage, specific assessment of timing of the traumatic event, and prospective designs could help mitigate this issue.

A second challenge for developmental research aiming to define predictors for PTSD risk is the need for large cohort studies which prospectively assess neural structure and function and trauma exposures over development. Only one prospective study to date has collected MRI data prior to trauma exposure and onset of PTSD when the cohort happened to be exposed to the Boston marathon terrorist attack [15]. Longitudinal MRI studies in at-risk cohorts of children growing up in inner city communities with high levels of violence exposure are underway and will be needed to better understand the relation between MRI abnormalities and PTSD risk. Additionally, large developmental MRI studies that include regular trauma assessments are needed to understand the role of brain structure and function in development of post-trauma psychopathology.

A final consideration for longitudinal developmental studies is related to the long-term consequences of stress and trauma and related brain alterations. It is postulated that traumatic events during development could shape brain structure and function to be adaptive in the current situation [115], but it is unclear whether these changes will be predictive of later PTSD or, alternatively, promote resilience. Furthermore, over time neural compensation could lead to previously overactive regions now showing decreased activation or possibly atrophy, which could be related to different psychiatric risk profiles. Therefore, continuing to monitor developmental cohorts and assessments of psychopathology in adulthood will be instrumental to elucidate the long-term effects of stress and trauma on the brain, which will provide potential targets to promote resilience early on during development.

## Summary and conclusions

Longitudinal neuroimaging studies of trauma and related mental health consequences are beginning to outline a consistent set of factors contributing to trauma resilience. The overall pattern across networks and timepoints is illustrated in Fig. 2. Engagement of response areas of the threat network has a consistent negative correlation with resilience, while threat inhibition-related areas, the reward network, and the cognitive control network have a consistent positive correlation. The picture is more complex after the trauma has occurred. The attention network exhibits a positive correlation with resilience post-trauma. However, changes across certain networks appear to support recovery over time. While the DMN engagement peri-trauma at first may be maladaptive, this early engagement predicts later recovery years post-trauma. In addition, pre-trauma salience activity has a negative association with resilience, but higher salience activation post-trauma predicts both peri-trauma resilience and later recovery. Further complicating the picture, there appear to be subgroups of people in which threat inhibition activity is negatively associated with resilience (e.g., the dissociative subtype of PTSD). Together, the emerging literature on longitudinal studies of trauma suggests that stable individual differences in core affective regions predict resilience, whereas higher-order associative networks flexibly adapt following trauma, with an initial tradeoff for early DMN engagement followed by long-term benefit.

These conclusions, however, must be tempered by the fact that this is a relatively new literature, with a number of limitations. Studies of the pre-trauma period are particularly sparse, with very small samples and power-related limits on more exploratory analyses. Future work in larger samples needs to address neural circuit function across the brain, looking at relationships between pre-trauma circuit-level connectivity and psychological outcomes post-trauma, as well as changes in these circuits following trauma. Such studies may very well reveal pre-trauma resilience factors outside the threat and salience networks. Additionally, biomarkers of resilience will only become useful in prognosis and precision medicine approaches with further work to establish measurement variability, reliability, and expected normative values. This has been a major stumbling block for efforts to integrate neuroscience and psychiatry, and must become a key priority area for further research.

With these limitations in mind, we can leverage current findings to guide resilience-boosting interventions targeting different time windows and subgroups. For example, a recent study took a random selection of first-year college students to receive an intervention designed to increase their resilience through training in mindfulness, goal-building, and resilience skills [116]. For this type of intervention, in which the approach is broad, without respect to particular trauma or single stressful event, neurobiological targets might include improving modulation of the threat and salience network. In contrast, for studies in which the goal is to identify trauma exposed individuals shortly after the trauma, such as during an ED visit or in the battlefield, interventions may take an alternative approach of increasing the engagement of the dlPFC, inhibitory threat areas, or the attention networks. Moreover, in studies targeting individuals with chronic dissociative symptoms, interventions may instead attempt to decrease engagement of threat-inhibition regions. Due to changes across the DMN post-trauma and the limited data in respect to the reward network areas, further work is necessary to form new interventions regarding these areas. Future longitudinal work, taking into account potential network changes across pre-, peri-, and post-trauma, will provide critical data that may inform improved interventions that promote resilience.
